# Tissue factor and its procoagulant activity on cancer‐associated thromboembolism in pancreatic cancer: Comment by Mackman et al.

**DOI:** 10.1111/cas.15276

**Published:** 2022-02-07

**Authors:** Nigel Mackman, Yohei Hisada, Sierra J. Archibald

**Affiliations:** ^1^ UNC Blood Research Center Division of Hematology Department of Medicine University of North Carolina at Chapel Hill Chapel Hill North Carolina USA

## Abstract

The Quantikine^®^ ELISA detects tissue factor in cell lysates and culture supernatants containing extracellular vesicles from tissue factor‐expressing cell lines but does not detect low levels of tissue factor antigen in plasma.

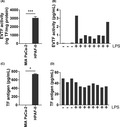

## DISCLOSURE

The authors have no conflict of interest.

Dear Editor,

Kobayashi et al.^1^ performed a prospective study to determine the relationship between levels of tissue factor (TF) and cancer‐associated thromboembolism in a Japanese cohort of pancreatic cancer patients. Four commercial human TF ELISA kits were used: IMUBIND^®^ (Sekisui Diagnostics), ZYMUTEST™ (Hyphen BioMed), Human TF ELISA (Abcam), and Quantikine^®^ ELISA (R&D Systems). These ELISAs were evaluated carefully for their ability to detect TF in cell lysates from multiple human cancer cell lines that express different levels of TF using western blotting with the anti‐human TF monoclonal antibody 10H10.^1^ Based on the level of the signal and linear regression analysis with the western blot data the authors chose to use the Quantikine^®^ kit for measurement of TF antigen in plasma from healthy controls and patients with cancer. They found that the median plasma levels of TF antigen signal were 39.1 pg/mL (range 16.8‐87.3) for healthy individuals, 47.2 pg/mL (range 0.0‐224.8) for patients with cancer who did not develop thrombosis, and 56.0 pg/m/L (range 37.6‐318.7) for patients who developed thrombosis.^1^ Multivariable analysis was used to show that the plasma TF antigen signal was an independent predictor of thrombosis in the patients with pancreatic cancer.

Measurement of TF antigen levels has been found to be problematic when using commercial ELISAs due to the high background signal in plasma that is not observed for cell lysates or culture supernatant samples containing extracellular vesicles (EVs) from cell lines.^2^ For instance, a wide range of values (between 61 and 187 pg/mL) has been reported in different studies using the IMUBIND^®^ ELISA for TF antigen signals in plasma from healthy individuals.^3^ This may be in part due to differences in the plasma preparation. Parhami‐Seren et al.^3^ used the IMUNBIND^®^ ELISA with their own calibrator to measure TF‐like antigen in the plasma of 91 healthy individuals and found that the majority (72 individuals) had levels below the quantitative limit of the assay (65.4 pg/mL), 6 had levels between 65.4 and 163.5, and in 13 individuals the non‐specific signal was higher than the specific signal.

Ourselves and others use plasma prepared from whole blood with or without LPS stimulation of monocytes as positive (containing TF + EVs) and negative controls, respectively, for TF antigen and activity assays. We used an in‐house extracellular vesicle tissue factor (EVTF) activity assay to measure the levels of TF + EVs in samples and observed a large increase in signal in the positive controls compared with the matched negative controls.^4^, ^5^ In 1 study, we found that 6 healthy individuals had a high TF antigen signal level (263.9 ± 32.9 pg/mL) in the negative control plasma using the IMUBIND^®^ ELISA with the standard from the kit.^4^ There was a small increase (1.4 ± 0.3‐fold) in levels of TF antigen signal in 5 out of 6 individuals in the matched positive controls compared with the negative controls; this was far smaller than the increase in EVTF activity. The ZYMUTEST^™^ ELISA gave a low signal (8.8 ± 1.9 pg/mL) in the negative controls, but there was no increase in the positive controls.

Kobayashi et al.^1^ used the Quantikine^®^ ELISA. Interestingly, the Quantikine^®^ ELISA was used to measure plasma TF antigen levels in 805 patients in the Comparison of Acute Treatments in Cancer Hemostasis (CATCH) trial for patients with cancer.^6^ The authors concluded that TF antigen was predictive of recurrent venous thromboembolism.^6^ In our study prior to the CATCH study, we had evaluated the ability of Quantikine^®^ ELISA to measure levels of TF antigen in plasma.^7^ We observed a wide range of values for negative control plasma from 5 donors (20.2 [4.9‐77.2]. median, interquartile range), which was slightly lower than the value reported by Kobayashi et al. (39.1 [16.8‐87.3]).^1^ However, as seen with the ZYMUTEST^™^ ELISA, there was no increase in the signal in positive controls compared with negative controls with this ELISA. We wrote an “understanding the pathway” article on the study by Khorana et al.^6^ and concluded that the Quantikine^®^ ELISA does not measure TF antigen in plasma.^2^

**FIGURE 1:**
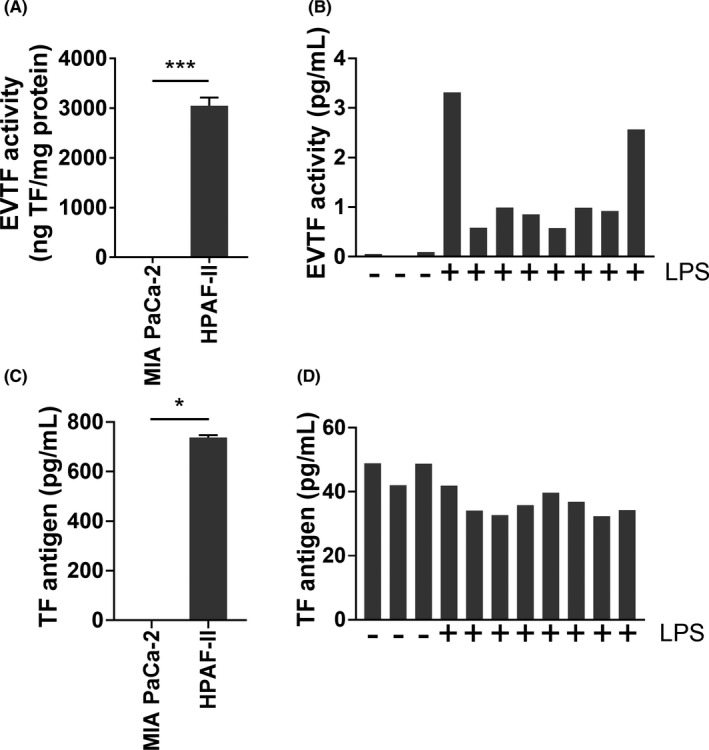
Measurement of extracellular vesicle tissue factor (EVTF) activity and TF antigen in culture supernatants and plasma. A, Levels of EVTF activity in culture supernatant of MIA PaCa‐2 and HPAF‐II cell lines. Values were normalized to the protein concentration of EVs. Data are shown as mean + standard deviation (measured in triplicate). Welch *t* test was used based on the distribution and variance determined with the Shapiro‐Wilk test and the F test. ***P < .001. B, Levels of EVTF activity in plasma prepared from whole blood treated with or without LPS. Three plasma samples were prepared from whole blood treated without LPS treatment (LPS−) and 8 plasma samples were prepared from whole blood treated with LPS treatment (LPS+). C, Levels of TF antigen in culture supernatants of MIA PaCa‐2 and HPAF‐II cell lines. Data are shown as median + interquartile range (measured in triplicate). Mann‐Whitney *U* test was used based on the data distribution determined with the Shapiro‐Wilk test. *P < .05. D, Levels of TF antigen in plasma prepared from whole blood treated with or without LPS

Here, we assessed the ability of the Quantikine^®^ ELISA to detect TF antigen in cell supernatant samples containing EVs from TF‐positive and TF‐negative human pancreatic cancer cell lines, and in plasma samples from whole blood with or without LPS stimulation.^8^ We observed high levels of EVTF activity with the TF‐expressing cell line (HPAF‐II) and the LPS‐treated plasma but not with the TF‐negative cell line (MIA PaCa‐2) and the untreated plasma (Figure 1A,B). Consistent with the results from Kobayashi et al.^1^ using cancer cell lines, the Quantikine^®^ ELISA gave a strong signal with the TF‐positive cell line, HPAF‐II, and no signal with the TF‐negative cell line, MIA PaCa‐2 (Figure 1C). However, unlike the EVTF activity data, we did not observe any difference between the signal in the untreated plasma samples compared with the LPS‐treated plasma samples (Figure 1D). We believe that this is most likely to be due to a high non‐specific signal masking the specific signal for TF antigen. In summary, the Quantikine^®^ ELISA detects TF in cell lysates and culture supernatants containing EVs from TF‐expressing cell lines, but does not detect low levels of TF antigen in plasma.

## References

[1] Kobayashi S , Koizume S , Takahashi T , et al. Tissue factor and its procoagulant activity on cancer‐associated thromboembolism in pancreatic cancer. Cancer Sci. 2021;112:4679‐4691. 34382298 10.1111/cas.15106 PMC8586686

[2] Ay C , Mackman N . Tissue factor: catch me if you can! J Clin Oncol. 2017;35:1128‐1130. 28029321 10.1200/JCO.2016.70.6788

[3] Parhami‐Seren B , Butenas S , Krudysz‐Amblo J , et al. Immunologic quantitation of tissue factors. J Thromb Haemost. 2006;4:1747‐1755. 16879217 10.1111/j.1538-7836.2006.02000.x

[4] Lee RD , Barcel DA , Williams JC , et al. Pre‐analytical and analytical variables affecting the measurement of plasma‐derived microparticle tissue factor activity. Thromb Res. 2012;129:80‐85. 21737126 10.1016/j.thromres.2011.06.004 PMC3272762

[5] Hisada Y , Mackman N . Measurement of tissue factor activity in extracellular vesicles from human plasma samples. Res Pract Thromb Haemost. 2019;3:44‐48. 30656275 10.1002/rth2.12165 PMC6332748

[6] Khorana AA , Kamphuisen PW , Meyer G , et al. Tissue factor as a predictor of recurrent venous thromboembolism in malignancy: biomarker analyses of the CATCH trial. J Clin Oncol. 2017;35:1078‐1085. 28029329 10.1200/JCO.2016.67.4564

[7] Claussen C , Rausch AV , Lezius S , et al. Microvesicle‐associated tissue factor procoagulant activity for the preoperative diagnosis of ovarian cancer. Thromb Res. 2016;141:39‐48. 26967531 10.1016/j.thromres.2016.03.002

[8] Rosell A , Moser B , Hisada Y , et al. Evaluation of different commercial antibodies for their ability to detect human and mouse tissue factor by western blotting. Res Pract Thromb Haemost. 2020;4:1013‐1023. 32864552 10.1002/rth2.12363 PMC7443430

